# SUPPORTING AGING IN PLACE THROUGH NEW MOBILITY SOLUTIONS

**DOI:** 10.1093/geroni/igae098.1576

**Published:** 2024-12-31

**Authors:** Fatemeh Hatami, Anu Siren, Steve O’Hern

**Affiliations:** Tampere University, Tampere, Pirkanmaa, Finland; Tampere University, Tampere, Pirkanmaa, Finland; University of Leeds, Leeds, England, United Kingdom

## Abstract

Aging in place is among the most widespread contemporary policy concepts. However, an important prerequisite for aging in place is the possibility to attend out-of-home activities, which requires satisfactory mobility options. Previous research has indicated that new transportation solutions and technologies may hold promise for older people’s mobility needs. To investigate these promising opportunities and how they may support aging in place, a critical literature review was conducted. Using Web of Science, Scopus, and PubMed as electronic journal databases, we searched keywords representing the user group (older adults), the solution (transport systems), and its qualities (novel), limiting the search to publications between 1990-2023. The search was complemented by hand-searching of reference lists and selected project homepages. The initial search yielded more than 3000 publications. After screening for title, abstract and contents, around 110 publications were included for review. To create a conceptual model, we synthetized major findings, and identified common themes, trends, and disparities in the existing literature. We analyzed whether proposed solutions are evidence based and whether the literature focuses on implementation processes. We also analyzed which mobility needs the solutions are explicitly targeting, and which groups of older adults are thought to be the main users of these solutions. Our conclusion is that while literature addresses many novel solutions that have potential for supporting older adults’ mobility, there are significant knowledge gaps related to assessing the feasibility of these solutions.

